# Barricades and brickwalls – a qualitative study exploring perceptions of medication use and deprescribing in long-term care

**DOI:** 10.1186/s12877-016-0181-x

**Published:** 2016-01-15

**Authors:** Anna Palagyi, Lisa Keay, Jessica Harper, Jan Potter, Richard I. Lindley

**Affiliations:** The George Institute for Global Health, Sydney Medical School, University of Sydney, Sydney, NSW Australia; Illawarra Health and Medical Research Institute, University of Wollongong, Wollongong, NSW Australia; Illawarra-Shoalhaven Local Health District, Wollongong, NSW Australia

**Keywords:** Long-term care, Medication review, Polypharmacy, Deprescribing, Medication

## Abstract

**Background:**

The co-administration of multiple drugs (polypharmacy) is the single most common cause of adverse drug events in the older population, and residents of long-term care facilities (LTCFs) are at particularly high risk of medication harm. ‘Deprescribing’ – the withdrawal of an inappropriate medication with goal of managing polypharmacy and improving outcomes – may improve the quality of life of LTCF residents. The RELEASE study sought to explore perceptions of medication use and the concept of deprescribing in LTCFs.

**Methods:**

Focus groups and interviews were conducted with General Practitioners (GPs), pharmacists, nursing staff, residents and their relatives within three LTCFs in the Illawarra-Shoalhaven region of NSW, Australia. Audiotapes were transcribed verbatim and, using the Integrative Model of Behaviour Prediction as a framework, thematic analysis of transcripts was conducted using QSR NVivo 10.

**Results:**

Participants acknowledged the burden of too many medications (time to administer, physical discomfort, cost), yet displayed passivity towards medication reduction. Residents and relatives lacked understanding of medicine indications or potential harms. Willingness to initiate and accept medication change was dependent on the GP, who emerged as a central trusted figure. GPs preferred ‘the path of least resistance’, signalling systems barriers (poor uniformity of LTCF medical records, limited trained LTCF personnel); time constraints (resident consultations, follow-up with specialists and family); and the organisation of care (collaborating with LTCF staff, pharmacists and prescribing specialists) as obstacles to deprescribing.

**Conclusions:**

Targeted engagement is required to raise awareness of the risks of polypharmacy in LTCFs and encourage acceptance of deprescribing amongst residents and their relatives. GPs are integral to the success of deprescribing initiatives within this sector.

**Electronic supplementary material:**

The online version of this article (doi:10.1186/s12877-016-0181-x) contains supplementary material, which is available to authorized users.

## Background

Older people in Australia accumulate large numbers of different medications, often prescribed by a number of specialists as well as the General Practitioner (GP). A cross-sectional survey of 4500 community-dwelling Australians found that almost 50 % of respondents aged 65 years and older reported taking five or more medicines, and more than 10 % reported using 10 or more [1]. For residents of long-term care facilities (LTCFs), a frail population with often complex health conditions, the average number of prescribed medications is significantly higher than their community-dwelling counterparts [2, 3]. Studies in the long-term care setting report polypharmacy (defined as the concomitant use of five or more medicines) in up to 95 % of residents, with an average of 7–10 medications per resident [3–5].

The use of greater numbers of medications has been independently associated with an increased risk of adverse drug events in both community-dwelling [6] and institutionalised [7] older persons, as well as an increased risk of hospital admission [8]. A review of Australian adverse drug event data [9] identified that more than 30 % of unplanned hospital admissions for people over 75 years of age were medication related. For residents of LTCFs, adverse drug reactions are common [7, 10, 11] and resulting hospital admissions often lend greater complexity to a resident’s medication regimen [12, 13]. While admission to hospital might provide an opportunity for medication review, analysis of over 200 patients aged 70 years and older discharged from acute care hospitals to LTCFs in Brisbane and Melbourne, Australia, showed that the proportion of these patients taking four or more potentially inappropriate medicines (PIMs) actually increased from 0.5 % on admission to 2.9 % on discharge [12].

Deprescribing – the process of withdrawal of an inappropriate medication supervised by a health care professional with the goal of managing polypharmacy and improving outcomes [14] – is a health care innovation with a real potential to relieve unnecessary medication-related suffering and disability in vulnerable older populations [15]. In the long-term care setting, efficacious deprescribing may not only have a positive impact on residents’ physical and social functioning, but also make available valuable resources for the provision of timely and appropriate care addressing the physical, spiritual, social and cultural needs of each resident [16]. A number of tools exist to assist practitioners in deprescribing decisions [17–22], and feasibility studies in both community-dwelling [23–25] and facility-based [26, 27] adults have suggested that the concept is acceptable to patients and recruitment feasible. While clinical trials of the safety and efficacy of medication withdrawal in the older population remain in their infancy, studies to-date have demonstrated both short-term physical and cognitive benefits, and no significant harm, following reduction of antihypertensives, benzodiazepine, psychotropic agents and statins [28, 29].

While the impact of deprescribing will depend on both the clinical implications and patient acceptance of medication withdrawal, few studies have implemented qualitative methods to explore perception of medication use and discontinuation held by either patient or health professional [25, 30]. Further, institutionalised persons such as hospital inpatients and residents of LTCFs have up to now been largely omitted from research in this field. Common barriers and enablers influencing a decision to cease a medication have been reported by community-based patients of varying ages [31]. These include a perceived benefit of medicine continuation, the time and physician support required during the process of deprescribing, the influence (both positive and negative) of family and health professionals, and a fear of adverse effects from medicine cessation.

The RELEASE study – *Dep***r***escribing: A n***e***w affordable health care mode***l***for the pr***e***scribing and***a***dministration of medication***s***for vulnerable older people in ag***e***d care homes* – is a qualitative study undertaken to explore a range of key stakeholder perspectives on medication use and the concept of deprescribing in the Australian long-term care sector. RELEASE aims to improve our understanding of the attitudes towards medication reduction held by the frail elderly in residential care – a population group taking more medications than any other, yet who have been underrepresented in studies of deprescribing to-date. This paper reports perceptions of medication use and the concept of deprescribing for LTCF residents, as identified by the RELEASE study participants. The application of these findings to informing the development of deprescribing initiatives within the aged care sector is discussed.

## Methods

We conducted focus groups with GPs, staff members, residents and their relatives within LTCFs in the Illawarra-Shoalhaven region of New South Wales. Semi-structured interviews with pharmacists, all providing pharmaceutical dispensing or review services to LTCFs within the same region, were also undertaken by a member of the research team.

Participating GPs were recruited by newsletter announcements and email invitation distributed by the Illawarra-Shoalhaven Medicare Local – a local, not-for-profit organisation supporting the primary health sector. The Director of Nursing at each collaborating LTCF initially nominated residents considered able to provide informed consent and participate in focus group discussions, and these individuals were then approached separately by the study Geriatrician who assessed cognitive and communicative capacity. Only those residents with mental capacity to understand the study were invited to take part. LTCF staff and relatives of residents were invited to take part in the study’s focus groups by notices placed in common areas of the facilities and direct invitation from the facility manager. Two participating pharmacists were approached directly by a member of the research team and snowball sampling [32] used to recruit additional pharmacists. All participants provided informed written or oral consent.

**Fig. 1:**
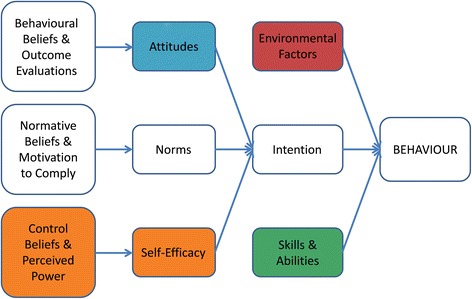
An Integrative Model of Behaviour Prediction, adapted from Fishbein (2006). Themes emerging from the analysis were most clearly associated with five key elements: environmental factors, attitudes, control beliefs and self-efficacy, and skills and abilities

Focus groups with residents, their relatives and LTCF staff were conducted on site at each collaborating LTCF to ensure that participants were in a comfortable environment to facilitate an open discussion. The GP focus group was held at a breakfast meeting at the Medicare Local headquarters. Discussion guides unique to each participant group were developed based on the framework of the Integrative Model of Behaviour Prediction (IMBP) [33] (Fig. 1) and used to prompt dialogue according to the model’s key constructs. The IMBP is a theoretical model used widely in health behaviour research which predicts that people act on their intentions when they have the necessary skills and when environmental factors do not impede behavioural performance [34]. Our discussion guide topics included current behavioural practices and normative beliefs regarding prescription medication use by older LTCF residents; understanding of and attitudes towards deprescribing; external factors influencing medication behaviour; and potential approaches to a deprescribing strategy within the aged care sector (Additional file 1). Each focus group was conducted in English and led by a moderator with a health research background and experience as an interviewer and group facilitator (LK/AP), with one observer and note taker (JH). A summary of the discussion was read back to participants at the completion of each focus group in order to confirm the interpretations of the participants’ responses made by the moderator. These focus groups were audiotaped and lasted approximately one hour. Basic demographic details of focus group participants were elicited by a short, interview-based questionnaire.

Semi-structured interviews with pharmacists were conducted via telephone and aided by an interview guide also developed using the framework of the IMBP (Additional file 1). An overview of the professional experience of participating pharmacists was obtained. All interviews were audiotaped.

Ethics approval for the RELEASE study was granted by the University of Sydney Human Research Ethics Committee (Project No. 2013/1076). Data collection was carried out between January and October 2014.

### Data analysis

Focus group audiotapes were transcribed verbatim and the transcript checked for accuracy by a member of the research team. Using QSR NVivo 10 (QSR International, Burlington, MA, USA), transcripts were then separately coded by two members of the research team and codes reviewed, refined and sorted into a single coding framework based on main categories of discussion. Emerging themes were explored by all members of the research team in relation to the framework of the IMBP, focusing on the constructs of attitudes, norms, self-efficacy, environmental factors, intent, and skills and abilities. The research team worked collaboratively to further refine themes and compare findings according to each participant group.

## Results

### Study participants

Nine focus groups were conducted with residents (*n* = 25), relatives (n = 16) and LTCF staff members (n = 19) from three LTCFs in the Illawarra-Shoalhaven area (Facility [F] A, B and C), prior to achieving data saturation. A tenth focus group was held with 8 GPs, all of who provided regular medical care to residents of LTCFs within the study region. Four pharmacists – two dispensing pharmacists and two accredited medication review pharmacists – completed semi-structured interviews.

A summary of focus group participants is provided in Table 1.

**Table 1:** Summary of participants of the RELEASE Study focus groups

	Facility A (96 residents)	Facility B (108 residents)	Facility C (100 residents)
Residents (n)	13	7	5
Age (mean, range), *years*	89 (76–100)	84 (75–93)	90 (83–99)
Female *%*	100	71	60
Length of time in LTCF (range), *years*	1 − 13	1 − 9	1 − 3
Number medications daily (median, range)	8 (5–20)	9 (2–17)	5 (5–10)
Relatives (n)	5	7	4
Age (mean, range), *years*	63 (55–71)	70 (54–84)	63 (55–72)
Female *%*	40	83	75
LTCF staff (n)	8	7	4
Age (mean, range), *years*	45 (21–69)	40 (27–54)	51 (44–56)
Time working in aged care (range), *years*	1 − 15	3 − 20	2.5 − 8
General Practitioners (n)	8		
Age (mean, range), *years*	56 (43–73)		
Experience as GP (range), *years*	20 − 42		
Total LTCFs serviced (range)	1 − 6		
Total LTCF patients (range)	10 − 150		

*GP* general practitioner, *LTCF* long-term care facility

### Perceptions of medication use and deprescribing

**Fig. 2:**
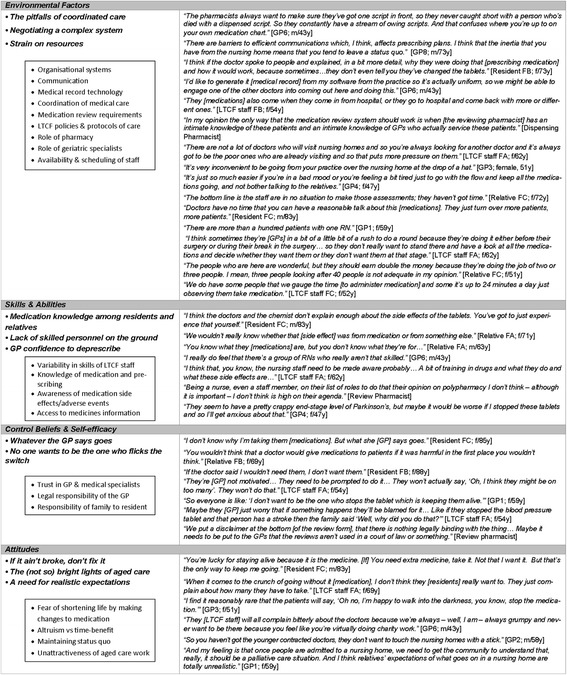
Major themes (in bold) and intermediate coding framework (boxed) emerging from discussions about medication use and deprescribing in long-term care facilities with participants of the RELEASE study. *GP* general practitioner, *f* female, *F* facility, *LTCF* long-term care facility, *m* male, *RN* registered nurse, *y* years

Although participant discussions were prompted to address all constructs of the IMBP framework, themes emerging from the analysis were most clearly associated with five key elements: environmental factors, attitudes, control beliefs and self-efficacy, and skills and abilities (Fig. 1). These are reported here and expanded in Fig. 2.

#### Environmental factors

Environmental factors were noted most frequently by all participant groups as influencing behaviours relating to medication use and medication management. These environmental influences included LTCF organisational systems (communication, medical record technology, coordination of a resident’s medical care), LTCF policies, the availability and scheduling of staff, staff workload burden, and coordinating the role of the various health care providers.

##### The pitfalls of coordinated care

It was noted that most frail older residents have a number of specialist healthcare providers in addition to their GP, and that inadequate communication between providers can cause inefficiencies in medication management. Some LTCF residents recounted having been prescribed medication for either an acute or circumstance-related condition in the past, e.g. an antibiotic, antihistamine, antidepressant, and, due to lack of review, this medication then being continued in their medication regime for months after the condition had subsided.

*“…so the psycho-geriatrician can come in and prescribe all these things and you come in two weeks later… you think, oh, I didn’t start that.”* [GP6; male, 43 years]

*“I’ve been taking Phenergan tablets for an itch. I have been on them two or three months, and I am taking three a day, and I don’t know what for. But I am still taking it.”* [Resident FA; female, 86 years]

The role of hospital admissions, both prior to a resident’s initial LTCF admission and during their stay, was highlighted by both LTCF staff and pharmacists as a significant contributor to medication accrual. Participants described how residents often amassed large numbers of new medications during hospital admission, prescribed according to hospital protocol. Following discharge back to the LTCF, residents then failed to undergo timely review of these new medications by their GP. One of the pharmacists expressed their concern, saying:

*“Often enough there's an intermediary system that plays a massive part in why they're on so much [medication]. It's a hospital… That's where the masses of medication get started and GPs, I feel, feel compelled to continue some of those medications.”* [Dispensing Pharmacist]

##### Negotiating a complex system

The organisation of a resident’s care within the LTCF emerged as an influential factor in medication management decisions. GPs in particular expressed frustration with communications arising from the LTCF, describing them as ‘relentless’ and ‘a never ending stream’, and were subsequently reluctant to engage in activity (such as ongoing medication refinement) that would inevitably require additional coordination with the LTCF.

*“I think negotiating with the nursing home to get things done is extremely frustrating, and I think that’s a big barrier for us to be able to get other doctors to actually provide services there.”* [GP6; male, 43 years]

An absence of standard medical charts within and across LTCFs also vexed GPs, adding to their perceived complexities of initiating medication change. A number of GPs expressed their preference for electronic medical records and the ability to use their practice software for their LTCF patient records.

*“When anybody comes and writes anything, they just squash out one and write underneath it and it ends up becoming like a dogs dinner… That drives me mad. So lack of uniformity of medication documentation is a barrier.”* [GP3; female, 51 years]

The present frequency and structure of resident medication reviews, conducted as part of the compulsory biennial LTCF Residential Medication Management Review (RMMR) program and at other times on request of the GP, was questioned by some participants. Pharmacists and relatives expressed concern that the current compulsory two year RMMR schedule is far too long.

*“Two years is not good enough really, to be honest with you, because as you know it’s an aged care facility and there’re a lot of things that go on in two years that need to get addressed in regard to medication.”* [Dispensing Pharmacist]

Medication reviews conducted under the RMMR program did not involve face-to-face assessment of individual residents but instead relied on a review of medication charts and resident notes. GPs stated that they were more likely to apply the recommendations of a medication review that was conducted by a local pharmacist with resident contact, rather than the automated RMMR report.

*“And do you have any personal interaction with the resident during the review process?”* [Facilitator] *“See, that’s the thing – not really, no… It’s always relied on staff notes, staff reports and all that they’re seeing in behaviour… and yeah, that’s the way it’s done.”* [Review Pharmacist]

*“I’d just rather someone like Pharmacist X or Pharmacist Y, or someone whose opinion we actually really respect [conduct the medication review]. When they do them they’re high quality and actually useful for the patient – rather than just having it stitched up by this cynical document which is computer generated.”* [GP2; male, 58 years]

##### Strain on resources

Time restraints and a shortage of health professionals willing to engage with aged care were cited by both LTCF staff and GPs as major obstacles to the implementation of GP-led medication reviews and consequential deprescribing strategies for individual residents. All participating GPs provided care to LTCF residents on a part time basis and experienced difficulties in balancing routine visits to the LTCF, responding to LTCF emergency requests and following up with a resident’s medical specialists and family, with their regular practice workload. The acknowledged additional time required to implement a strategic approach to deprescribing was a critical impediment to their willingness to engage in such a process. In Australia, GPs do not have a fixed practice list and difficulties in successfully promoting aged care amongst younger GPs meant that the bulk of the workload fell on the more senior GPs who had been engaged in LTCF visits for years.

*“All of our doctors are essentially part-time… And the challenge of the part-time GP workforce in covering nursing homes, providing on call services, is something that we have never managed to sort out, but have always hoped to.”* [GP3; female, 51 years]

*“It’s a great idea to reduce medication if you can do it in a safe manner that’s not going to make us have to go out to the nursing home 55 more times.”* [GP5; male, 67 years]

The shortage of registered nurses (RN) rostered for each LTCF shift and the high task-load of carers was a concern raised by family members, who felt that there was inadequate time available for LTCF staff to conduct the observation necessary to identify any medication-related issues of their relative. Some carers described spending “most of their shift” conducting medication rounds, and an average time of 2 h for each of the three daily medication rounds was experienced across all participating LTCFs.

*“I don’t think they’ve got time to observe whether there’s a change needed or they [medications] should be reviewed.”* [Relative FC; female, 72 years]

#### Skills and abilities

All participants questioned either their own or others’ knowledge of an older person’s medication requirements and ability to initiate indicated changes to a resident’s medication regimen.

##### Medication knowledge among residents and relatives

A lack of knowledge of medicine indications, actions and potential adverse events was evident in both LTCF residents and their relatives. This appeared to contribute to a general apathy towards polypharmacy and the concept of PIMs from both groups. While the majority of residents were aware of the number of pills they were required to take each day, they had little idea of the indication for individual medicines. Relatives held a similarly limited understanding – many described keeping track of medicine names and numbers through pharmacy invoices but again were unfamiliar with the specific medical indication for which each medication had been prescribed.

Both residents and relatives were able to identify only few medication side effects – ‘dry mouth’ and ‘drowsiness’ being the most common. Those with prior experience of a medication-related incident had some wider adverse event knowledge, including warfarin-related bleeding and dizziness. Overall, however, there was minimal recognition of medication-related adverse drug reactions, including the well-established increased risk of falls and impaired physical and cognitive function.

*“I take so many tiny little ones. And what they are for, I don’t know.”* [Resident FA; female, 71 years]

*“I don’t know what the negatives [medication side effects] would be, but sometimes they just flush through your body. They’re not really essential if you don’t physically work.”* [Relative FB; female, 71 years]

##### GP confidence to deprescribe

GPs varied in their confidence to make informed decisions about medication reduction and cessation. Medicines for Alzheimer’s disease, dementia and Parkinson’s disease posed particular uncertainty for GPs when considering a deprescribing strategy. GPs noted the absence of any protocol for deprescribing and agreed that standard protocols of medication management across LTCFs could facilitate co-ordinated medical decision making by all persons providing care to the resident.

*“I’m not confident about other medications though… Say someone was Parkinsonism drugs – I would be less confident stopping it because I do initiate anti-Parkinsonism drugs, but not at the higher end of them.”* [GP7; male, 52 years]

*“If there was some protocol-driven way [to deprescribe]… for example, teleconferencing with a geriatrician every time someone is admitted to a nursing home, something like that, to set up [a resident’s medication] protocol. Something that doesn’t really involve us as much.* [GP3; female, 51 years]

##### Lack of skilled personnel on the ground

A number of GPs complained about the skill deficiency of LTCF nursing staff, which they believed added unnecessarily to their burden of responsibility as well as complicating medical care processes. Pharmacists also commented on the need for the further education of nursing staff and carers, particularly in relation to medication side effects and their documentation and management. For one GP, the perceived higher level if skills displayed by a small number of nursing staff was the only thing keeping them engaged in the aged care sector.

*“… most [nursing staff] are very under skilled, very unintelligent, and not able to make any decisions for themselves.”* [GP; female, 59 years]

*“It’s a matter of educating them [nursing staff] to understand that they don’t need to know the medicines… they don’t need to know specific details, they need to provide information about that resident that’s documented well and correctly so that we can use that information.”* [Review Pharmacist]

#### Control beliefs & self-efficacy

Participants perceived varying external factors as restricting their ability to query, initiate or manage medication-related issues, and expressed limited belief in their own capabilities to independently instigate medication change. Together these factors appear to have created apathy, absence of accountability, and lack of confidence to initiate strategies of medication reduction for LTCF residents in this setting.

##### Whatever the GP says goes

The basis of the apathy towards medication use displayed by LTCF residents and relatives appeared to rest in their complete trust in the care and decisions of the GP. Participating LTCF residents, relatives, staff and pharmacists placed the GP as the pivotal entity in a resident’s prescribing decisions and ongoing medication management, and as holding the key to the success of any potential deprescribing strategy. While a number of LTCF staff questioned some GPs’ motivation and self-efficacy towards initiating medication review and modification, the overwhelming belief from residents and their relatives was that a GP’s management is not to be questioned. Present LTCF policies and procedures governing the process for a resident’s medicines decisions, where medication-related queries are escalated from carer to RN to GP reinforce the significance of the role of the GP.

*“The doctor has got the training and the knowledge so we have to go with their recommendation.”* [Relative FA; female, 67 years]

##### No one wants to be the one who flicks the switch

A medico-legal angle on deprescribing was broached by both GPs and pharmacists. GPs discussed exercising caution with initiating medication changes, particularly where they assumed a resident’s family had strong expectations of medicines keeping their relative alive. This perceived pressure from relatives served as a barrier to ceasing or reducing certain drug classes. GPs were also cautious with the documented recommendations of non-commissioned medication reviews – expressing concern about the legal risks of not implementing review recommendations. The participating review pharmacists had both experienced the averseness of GPs towards non-commissioned reviews but dismissed their legal concerns, confirming that the medication review report was not a legally binding document.

*“It’s [medication review report] one of those pieces of paper which goes in the shredder for me, if I haven’t got the time to negotiate or to really think about it. Because medico-legally it’s a document which actually stitches you up.”* [GP6; male, 43 years]

#### Attitudes

##### If it ain’t broke, don’t fix it

While the total numbers of daily medications for an individual resident was often viewed as excessive by both the resident and relatives, and the physical discomfort of taking medicines a source of frequent complaints, neither group were self-motivated to query and modify current medicines. By habitually placing their trust in the decisions of the GP, residents and relatives had minimal experience in discussing medications issues or questioning prescribing decisions. A number of residents expressed fear at the concept of reducing or ceasing some of their current medications, believing that their medicines were prolonging their life.

*“They’d [the GP] have to convince me that it was the right thing to do before I would agree to it.”* [Relative FB; female, 69 years]

*“That’s the only way I’m still walking on two legs. If I didn’t have it [medication] I’d be probably already six foot under the ground.”* [Resident FC; male, 83 years]

##### The (not so) bright lights of aged care

Participating GPs described the general perception among their profession of LTCF work being time-consuming and frustrating, and having negative financial impact on practice profits. Under current arrangements, GPs are reimbursed for LTCF consultations via the Government’s Medicare Benefits Schedule, within which per-patient reimbursement is lower when multiple consultations are conducted during a single visit, and there is no compensation for travel time and clinically relevant tasks required post visit. GP participants believed that these factors contributed to the overall disinterest in working in aged care, and were strong deterrents to graduate and younger doctors joining this aspect of medical practice. The lack of support for LTCF activity from the next generation of GPs meant that GPs currently servicing LTCFs were overwhelmed with day-to-day responsibilities, less likely to engage in time-consuming tasks such as medication reviews and deprescribing decisions, and generally preferring to maintain the status quo.

*“I’d be very, very happy to give that [LTCF visits] to anybody who wanted it, pay a significant amount of money to get that off my hands. I’m completely burnt out with it.”* [GP; female, 47 years]

##### A need for realistic expectations

There was some discordance between relatives’ and GPs’ perceptions of the role of the LTCF in the care of the resident. Many relatives viewed the LTCF as a 24 h medical care provider with staff on hand at all times for the needs of the resident. GPs, however, stressed a need for the community to accept the role of the LTCF as effectively a palliative care environment, and for relatives to be more realistic about the stage of life of their family member entering into LTCF care.

*“I think we really need to be in a situation where we’re educating relatives about what is realistic because it’s very hard to initiate the discussion with relatives. Look, why are we treating, why are we on all of these preventative medications when your relative is never going to get better and they are going to get progressively worse?”* [GP; female 59 years]

## Discussion

The RELEASE study is, to our knowledge, the first qualitative investigation of the perception of medication use and deprescribing in LTCFs by those individuals directly involved in the long-term care sector. While our findings reiterate key themes of time, knowledge, fear and acceptance reported in studies of acceptance of medication reduction elsewhere [31, 35], importantly this research emphasises the complexity of systems surrounding the long-term care population in relation to medication management: a cocktail of multiple healthcare providers, hospital admissions, rigid organisational structures, resource limitations, medical hierarchies, contrasting care expectations of family and GP and, amongst all of these, the life priorities of each individual resident.

It is evident here that, with the exception of medication review pharmacists, our participating stakeholders currently direct minimal attention to the identification and reduction of PIMs for LTCF residents. This is despite the ever increasing dialogue in the medical literature about the risks of polypharmacy in older patients and a need for deprescribing [15, 22, 35, 36], and helps explain the lack of progress in this matter. For the resident and their relatives, substantial trust in the advice and decisions of the GP allows for their reduced self-efficacy and general indifference about medication decisions. The integral role of the GP in this regard and their influence on patients’ understanding of medication benefits and acceptance of medication cessation has emerged in studies previously [25, 31, 35, 37]. In Australian LTCFs, where residents often have multiple specialist prescribing physicians, the role of the GP as the central coordinating practitioner is even more vital. The additional burden on the workload of GPs who provide aged care services has been emphasized by GP advocacy groups [38], with the non-contact time required to complete LTCF patient-related communications and documentation noted as both a resourcing and financial disincentive for GPs providing care for aged care residents.

System and resource related barriers to addressing medication issues for LTCF residents reported here are numerous, and for GPs these were a major deterrent to committing to the processes and engagement required for safe, rational medication reduction. The absence of a standardised electronic medical records system used across LTCFs, integrated with practice software, was highlighted as a significant hindrance to streamlined medication management for GPs currently servicing LTCFs and suggested as a deterrent to aged care work for the next generation of GPs. Communication challenges were raised by all participants – a consequence of the numerous stakeholders involved in the care of an older person residing in aged care. Communication has been previously highlighted as an enabler of successful medication cessation in community-dwelling patients [31, 37, 39–41]. The multiple layers of communication in LTCFs provide complexity to an individual’s care decisions and medication management: communication between resident and GP; between a resident’s various medical specialists; between relatives, nursing staff, hospital and GP; and between pharmacy and GP. The additional step of needing to talk to a surrogate decision maker for residents lacking mental competency make these discussions even more difficult. Issues arising from communication deficits have been previously linked to medication errors during a resident’s transition into a nursing home and following short-stay hospital admission [42].

Similar to findings of Spinewane et al. [43], participating GPs here expressed reluctance to interfere with medications that had previously been prescribed by a colleague or medical specialist. To some degree this was a result of lack of confidence in deprescribing skills, however an apparent fear of litigation, a perceived sense of responsibility to a resident’s family to “keep the resident alive”, and averseness to the additional workload required to initiate and manage medication change were also evident. Interestingly the value placed on a pharmacist’s recommendation for medication adjustments varied across individual GPs and was influenced by the length of working relationship between the two professionals. This finding supports previous studies which report that GPs are more likely to accept a pharmacist’s recommendations when there is personal rather than written conveyance of review outcomes [44] and when the review service is led by a pharmacist they know [45].

Hesitancy towards medication review and reduction generated by lack of knowledge and practise was exhibited across all stakeholders. Both residents and relatives highlighted their difficulty in obtaining medicines information from LTCF staff. Variability in the medication skills of LTCF care staff was a source of frustration for GP participants who believed it contributed to unnecessary communications from the LTCF and added to their workload. Pharmacists commented on the important role of LTCF staff in identifying and reporting potential adverse drug events, and suggested a need for upskilling in this area. Despite the availability of numerous tools to guide PIM identification and processes for medication cessation [17–22], GPs were largely unaware of standard protocols for deprescribing. These findings give impetus to the need for professional development on PIMs and deprescribing for physicians providing direct medical care for LTCF residents, as well as LTCF staff responsible for general daily care and monitoring.

While we have mapped a variety of perceptions of medication management, the conflicting views of relatives and GPs regarding of the role of medications in later life is perhaps one of the greatest attitudinal challenges. Death constitutes the primary reason for permanent LTCF resident separations each year (91 % cases), and one-quarter of these deaths occur within the first six months of admission [46]. GPs recognise that in this late stage of life medical care is at least partly palliative. In contrast, residents and their relatives maintain conviction in the benefits of medications for both longevity and quality of life, and relatives understandably struggle with the concept of end-of-life care and the potential loss of a loved one. Ensuring information about the goal of prescription medicines for the frail elderly and the potential harms of polypharmacy is available and accessible for residents and their relatives is prerequisite to encouraging their active involvement in shared decisions about medication change. Insufficient knowledge of the role of medications undoubtedly feeds fears of medication reduction and cessation.

### Limitations

This research was confined to aged care services in a single region in New South Wales, Australia and there are some limitations to the generalizability of findings to LTCFs and nursing homes in other settings. Although this study involved multiple stakeholders, the recruitment of participants was via self-selection and this may have resulted in a greater representation of individuals with stronger pre-existing attitudes towards medication use, particularly amongst participating residents and relatives. Further, barriers to the recruitment of GP participants limited our GP numbers to a single GP focus group, which (although rich in information) has consequences for the adequacy of representation of the spectrum of age and experience of GPs servicing LTCFs, as well as information saturation. Nevertheless, focus group discussions were conducted until we achieved saturation across all other stakeholder groups, indicating that the various themes were mostly well-explored and contrasting views elicited. Finally, there was limited cultural diversity across residents of participating facilities and further investigation of attitudes and beliefs towards medication reduction held by specific cultural groups and their health care providers is warranted.

## Conclusions

Our data suggests that the lack of progress in deprescribing in the aged care sector is due to a complex mix of reasons and therefore only a multi-faceted intervention addressing the multiple barriers identified here is likely to succeed. Such an intervention would need to address the operational systems in LTCFs, institute targeted engagement with residents and their support network, and recognise that GPs are key to any deprescribing initiative. The current systems of medical record keeping, communication and reimbursement in Australian long-term care are unfavourable to encouraging and rewarding a GP’s active engagement in PIM reduction. Deficits in knowledge and confidence of both LTCF nursing staff and GPs providing aged care services should be addressed to promote ongoing evaluation of a resident’s clinical need for deprescribing and improve self-efficacy in addressing PIM use. Without such efforts, the necessary motivation to overcome the multiple barriers to change may not be found.

## Additional file

Additional file 1:
**Focus Group Discussion Guide – Family/Relatives.** (PDF 260 kb)

## Abbreviations

GP: General practitioner
LTCF: Long-term care facility
PIM: Potentially inappropriate medicine
RELEASE: Dep**r**escribing: A n**e**w affordable health care mode**l** for the pr**e**scribing and **a**dministration of medication**s** for vulnerable older people in ag**e**d care homes
RN: Registered nurse
