# Supplementary material for: Barricades and brickwalls – a qualitative study exploring perceptions of medication use and deprescribing in long-term care
Source: BMC Geriatr. 2016 Jan 15;16:15. doi: 10.1186/s12877-016-0181-x (PMC4714480; doi:10.1186/s12877-016-0181-x)
Supplement: Additional file 1: — Focus Group Discussion Guide – Family/Relatives. (PDF 260 kb) [file 12877_2016_181_MOESM1_ESM.pdf]

FOCUS GROUP DISCUSSION GUIDE – FAMILY/RELATIVES

The following information will be collected for individual family members participating in a RELEASE study focus group.

Gender: Female / Male

Age: _______________

Length of your family member's LTCF residence: _______________

Number of different regular medications taken by your family member daily: ____________

Number of medical conditions of your family member: _______________

Indicative questions / discussion guide:

Questions to elicit behavioural beliefs

1. Tell me about your role in decision making with the medications taken by your family member/friend who is resident in the LTCF?
   - Do you attend medical appointments together?
   - How is it ensured that medicines are taken?
   - What is considered before prescribing medications for your family/friend?
2. Who do you think should be involved in decisions about reducing the number of medications taken by a resident?
   - Prompt: Resident, GP, pharmacist, nurse, family/carer, other.

Questions to elicit normative beliefs

3. How many different medications do you think is 'normal' for someone in an LTCF to take each day?
   - What do you consider a 'small' number of regular medications for a resident? And a large number?
4. Have you witnessed your family member/friend experience an event that you feel could be attributed to the medications they are taking?
   - What type of reactions have you seen? Prompt: fatigue, dizziness, falls, confusion.
   - What do you believe are the main risks for residents taking a large number of medications?

Questions to elicit control beliefs

5. How do you feel about the process of getting the right medicine for your family member/friend?
   - Are you able to get involved when you need to?

Questions to elicit attitudes

6. How do you feel about the medication use of your family member/friend?
   - Are you happy with the number of medications they are taking? Would you rather they take less medications?
   - Do you feel that their medication use is beneficial, or harmful?
   - What do you think about changes to the number of medications taken by older people in LTCF? Harms/benefits?

7. Generally, how do you think your family member/friend would respond if their GP told them that they could stop taking one of more of their medications?

Questions to elicit self-efficacy (belief in own ability to execute a task/change)

8. If you thought your family member/friend was not taking the right medicines for their health, what could you do in your role as family member or friend?
   - Would you do anything in the situation where you believed they were being adversely affected by their medications?
   - What would you do? Who would you talk to about this issue?
   - Do you feel confident about weighing up the risks and benefits of a particular medication yourself?
9. How involved would you say you are in decision-making generally for your family member/friend in the LTCF?
   - What types of issues are you involved in?
   - How much influence do you feel you have on the attitude of your family member/friend?

Questions to elicit environmental factors

10. Are you aware of any discussions within the LTCF about deprescribing for residents?
    - What discussions about medication use have occurred?
    - Have you been involved in any decisions regarding deprescribing and quality use of medicines for your family member/friend? Who else has been involved in these (LTCF management, pharmacy, external advocacy groups etc)?
11. How often does your family member/friend have the opportunity for contact with their GP or a pharmacist to discuss their medications?
    - Who normally initiates this? Are you present for these discussions?

Questions to elicit intent

12. Have you tried to get involved in decisions around the medications your friend/family member takes?
    - Prompt – prescribing decisions or ensuring they take medications

Questions to elicit skills and abilities

13. Do you consider the issue of medication costs?
    - How often? Tell me about these issues.
    - Do you think that the opportunity of paying for fewer medications would influence your willingness to have your family member/friend stop taking one of more of their medications?
14. How well do you understand the reasons why your family member/friend was prescribed each of their different medications?
    - Do you ever ask the nurse or GP for information about their medications?
    - Do you ask anyone else for information about their medications

FOCUS GROUP DISCUSSION GUIDE – LTCF NURSING STAFF

The following information will be collected for individual LTCF staff participating in a RELEASE study focus group.

Gender: Female / Male

Age: _______________

Years' experience as a health professional: _______________

Length of employment in current LTCF workplace: _______________

Length of previous employment in LTCF: _______________

Number of residents under your care in LTCF: _______________

Indicative questions / discussion guide:

Questions to elicit behavioural beliefs

1. Tell me about your role with the administering of medications to residents each day?
   - What is the normal routine?
   - What proportion of your work day is taken by the medicine rounds?
2. How do residents respond when it's time for their medication round?
3. Generally, how do you think a resident would respond if their GP told them that they could stop taking one of more of their medications?
4. Who do you think should be involved in decisions about reducing the number of medications taken by a resident?
   - Prompt: Resident, GP, pharmacist, nurse, family/carer, other.

Questions to elicit normative beliefs

5. How many different medications do you think is 'normal' for a resident of an aged care facility to take each day?
   - What do you consider a 'small' number of regular medications for a resident? And a large number?
6. Have you witnessed a resident experience an event that you feel could be attributed to the medications the resident is taking?
   - What type of reactions have you seen? Prompt: fatigue, dizziness, falls, confusion.
   - What do you believe are the main risks for residents taking a large number of medications?
7. Tell me about your main tasks day-to-day in the LTCF?
   - What proportion of your work day do you feel should be allocated to medicine rounds?
   - Are there tasks that you do not necessarily have time to do? Describe these?
   - What is preventing you from having time to do these tasks?

Questions to elicit control beliefs

8. What type of impact would it have on your day-to-day roles and responsibilities if the number of medications taken by residents was reduced?
   - Prompt: time/task allocation, responding to adverse events.

Questions to elicit attitudes

9. Have you thought about the possibility that some residents might be taken one or more medication that they do not necessarily need?
   - Would you do anything in the situation where you believed a resident was being adversely affected by their medications?
   - What would you do? Who would you talk to about this issue?
   - Do you feel confident about weighing up the risks and benefits to a resident of particular medication yourself?

Questions to elicit self-efficacy (belief in own ability to execute a task/change)

10. Do you have a voice in the medical management of residents?

Questions to elicit environmental factors

11. Have there been any discussions in your LTCF about deprescribing for residents?
    - What discussions about medication use have occurred?
    - Are you currently involved in implementing any strategies for deprescribing and quality use of medicines? Who is involved in these (LTCF management, pharmacy, external advocacy groups etc).
12. Discuss how involved the family/carers of residents are in decision-making for residents of your LTCF?
    - What types of issues would they be involved in?
    - How much influence do you feel they have on the attitude of the resident?
13. How often does a resident have the opportunity for contact with their GP or a pharmacist to discuss their medications?
    - Who normally initiates this?

Questions to elicit intent

14. Do you intend to speak with other people about medical management – who would this be?

Questions to elicit skills and abilities

15. How often would you explain to residents the reasons why they were prescribed each of their different medications?
    - Do residents ever ask for information?
    - Do you feel comfortable providing information about medicines?
    - Would you ever follow up with the pharmacist or resident's GP for information about a particular resident's medications?

16. Does the issue of medication costs arise in the LTCF?
    - How often? Tell me about these issues.
    - Do you think that the opportunity of paying for less medications would influence a resident's (and their family/carer's) willingness to stop taking one of more of their medications?

FOCUS GROUP DISCUSSION GUIDE – GENERAL PRACTITIONERS

The following information will be collected for individual GPs participating in the RELEASE study focus group.

Gender: Female / Male

Age: _______________

Years' experience as a GP: _______________ Practice location: _______________

Number of LTCFs serviced: _______________

Number of current patients resident in an LTCF: _______________

Proportion of current patients 65 years or older: _______________

Indicative questions / discussion guide:

Questions to elicit behavioural beliefs

1. Tell me about your role with the prescribing of medications to residents of aged care facilities?
   - What is the normal routine – do you visit the facility/does the resident visit your practice?
   - What proportion of your work day is taken by consultations with residents? And older people more generally?
2. Who do you think should be involved in decisions about reducing the number of medications taken by a resident of an aged care facility?
   - Prompt: Resident, GP, pharmacist, nurse, family/carer, other.
3. Have you previously approached the issue of poly-pharmacy with a resident? Generally, how do residents respond when you discuss the option of stopping one of more of their medications?
   - Any difference between residents and older people in the community?
   - Do you work with any other clinicians involved in the resident's care in order to change their medication regimen?

Questions to elicit normative beliefs

4. How many different medications do you think is 'normal' for a resident of an aged care facility to take each day?
   - What do you consider a 'small' number of regular medications for a resident? And a large number?
5. How commonly do you see a resident who has experienced an event that you feel could be attributed to the medications the resident is taking?
   - What type of reactions have you seen? Prompt: fatigue, dizziness, falls, confusion.
   - What do you believe are the main risks for residents taking a large number of medications?
6. What, in your opinion, would be the easiest and most useful drug classes to stop if you were to initiate deprescribing in LTCF residents?

Questions to elicit control beliefs

7. Discuss you general view of medication use in LTCFs.
8. What type of impact would it have on your day-to-day practice if the number of medications taken by residents of aged care facilities was reduced?
   - Prompt: writing of scripts, time/task allocation, responding to adverse events.
9. What do you think would be the biggest barrier to reducing medication in residents of LTCF?

Questions to elicit self-efficacy (belief in own ability to execute a task/change)

10. Have you thought about the possibility that some residents might be taking one or more medication that they do not necessarily need?
    - Would you do anything in the situation where you believed a resident was being adversely affected by their medications?
    - What would you do? Who would you talk to about this issue?
    - Is there any resistance to you making these changes?

Questions to elicit environmental factors

11. Have you been involved in any discussions with LTCFs about deprescribing for residents?
    - What discussions about medication use have occurred?
    - Are you currently involved in implementing any strategies for deprescribing and quality use of medicines? Who is involved in these (LTCF management, pharmacy, external advocacy groups etc).
12. Discuss how involved the family/carers of residents are in decision-making for residents of your LTCF?
    - What types of issues would they be involved in?
    - How much influence do you feel they have on the attitude of the resident?
13. How often does a resident or resident's family/friend have the opportunity for contact with their GP or a pharmacist to discuss their medications?
    - Who normally initiates this?
14. Do you consider the issue of medication costs for residents of an LTCF?
    - Do you think that the opportunity of paying for less medications would influence a resident's (and their family/carer's) willingness to stop taking one of more of their medications?

Questions to elicit intent

15. Discuss your views on the evidence for medication benefit for residents of LTCF.
    - Prompt: Pros/cons of poly-pharmacy; risks and benefits.
16. Is reducing the number of medications a consideration when treating a resident of LTCF?

Questions to elicit skills and abilities

17. Do you feel confident about weighing up the risks and benefits to a resident of particular medication yourself?
    - How do you take into consideration the medication advice provided by a Specialist practitioner? Would you be confident in declining their advice?
    - What support would you need to make a decision regarding stopping a particular medication?

INTERVIEW SCHEDULE – LTCF PHARMACISTS

The following information will be collected for key stakeholders interviewed as part of the RELEASE study.

Gender: Female / Male

Occupation / Organisation: _______________

Position: _______________

Indicative interview questions:

Questions to elicit behavioural beliefs

1. Tell me about your role with the administering of medications to residents of aged care facilities?
   - What proportion of your work day is taken by filling scripts for residents? And older people more generally?
2. Who do you think should be involved in decisions about reducing the number of medications taken by a resident of an aged care facility?
   - Prompt: Resident, GP, pharmacist, nurse, family/carer, other.

Questions to elicit normative beliefs

3. How many different medications do you think is 'normal' for a resident of an aged care facility to take each day?
   - What do you consider a 'small' number of regular medications for a resident? And a large number?
4. How commonly do you believe residents experience an event that you feel could be attributed to the medications the resident is taking?
   - What type of reactions have you seen? Prompt: fatigue, dizziness, falls, confusion.
   - What do you believe are the main risks for residents taking a large number of medications?
5. What, in your opinion, would be the easiest and most useful drug classes to stop if you were to initiate deprescribing in LTCF residents?

Questions to elicit control beliefs

6. What is your general view of medication use in LTCFs?
7. What type of impact would it have on your day-to-day tasks if the number of medications taken by residents of aged care facilities was reduced?
   - Prompt: filling scripts, time/task allocation.
8. What do you think would be the biggest barrier(s) to reducing medication in residents of LTCF?

Questions to elicit self-efficacy (belief in own ability to execute a task/change)

9. Have you thought about the possibility that some residents might be taken one or more medication that they do not necessarily need?
   - Would you do anything in the situation where you believed a resident was being adversely affected by their medications?
   - What would you do? Who would you talk to about this issue?

Questions to elicit environmental factors

10. Have you been involved in any discussions with/in your LTCF(s) about deprescribing for residents?
    - What discussions about medication use have occurred?
    - Are you currently involved in implementing any strategies for deprescribing and quality use of medicines? Who is involved in these (LTCF management, GPs, pharmacy, external advocacy groups etc).
11. Generally, how involved are the family/carers of residents in decision-making?
    - What types of issues would they be involved in?
    - How much influence do you feel they have on the attitude of the resident?
12. How often does a resident have the opportunity for contact with their GP or a pharmacist to discuss their medications?
    - Who normally initiates this?
13. Does the issue of medication costs arise in the LTCF?
    - How often? Tell me about these issues.
    - Do you think that the opportunity of paying for less medications would influence a resident's (and their family/carer's) willingness to stop taking one of more of their medications?

Questions to elicit intent

14. Discuss your views on the evidence for medication benefit for residents of LTCF.
    - Prompt: Pros/cons of polypharmacy; risks and benefits.

Questions to elicit skills and abilities

15. Do you feel confident in initiating changes to the way medicines are prescribed for LTCF residents?
    - What support would you need to make effective changes in reducing harmful poly-pharmacy for residents? Which individuals/groups would you like to see involved?
